# Dexamethasone treatment influences tendon healing through altered resolution and a direct effect on tendon cells

**DOI:** 10.1038/s41598-024-66038-5

**Published:** 2024-07-03

**Authors:** Franciele Dietrich-Zagonel, Md Abdul Alim, Leo Bon Beckman, Pernilla Eliasson

**Affiliations:** 1 Department of Biomedical and Clinical Sciences, Faculty of Medicine and Health Science, Linköping University, 581 83 Linköping, Sweden; 2 Department of Orthopaedics, Sahlgrenska University Hospital, Länsmansgatan 28, 431 80 Mölndal, Sweden; 3 Division of Immunology, Department of Pathology, University of Cambridge, Cambridge, UK

**Keywords:** Achilles tendon, Injury, Loading, Corticosteroids, Resolution, In vitro, Regenerative medicine, Experimental models of disease, Medical research, Translational research, Inflammation

## Abstract

Inflammation, corticosteroids, and loading all affect tendon healing, with an interaction between them. However, underlying mechanisms behind the effect of corticosteroids and the interaction with loading remain unclear. The aim of this study was to investigate the role of dexamethasone during tendon healing, including specific effects on tendon cells. Rats (*n* = 36) were randomized to heavy loading or mild loading, the Achilles tendon was transected, and animals were treated with dexamethasone or saline. Gene and protein analyses of the healing tendon were performed for extracellular matrix-, inflammation-, and tendon cell markers. We further tested specific effects of dexamethasone on tendon cells in vitro. Dexamethasone increased mRNA levels of S100A4 and decreased levels of ACTA2/α-SMA, irrespective of load level. Heavy loading + dexamethasone reduced mRNA levels of FN1 and TenC (*p* < 0.05), while resolution-related genes were unaltered (*p* > 0.05). In contrast, mild loading + dexamethasone increased mRNA levels of resolution-related genes ANXA1, MRC1, PDPN, and PTGES (*p* < 0.03). Altered protein levels were confirmed in tendons with mild loading. Dexamethasone treatment in vitro prevented tendon construct formation, increased mRNA levels of S100A4 and decreased levels of SCX and collagens. Dexamethasone during tendon healing appears to act through immunomodulation by promoting resolution, but also through an effect on tendon cells.

## Introduction

The treatment approach for acute Achilles tendon rupture remains a subject of debate^1^, highlighting the importance of comprehending tendon healing mechanisms, with or without specific treatments. After injury, the inflammatory phase initiates the healing cascade, followed by overlapping proliferative and remodeling phases^2^. In conjunction with the onset of inflammation, a resolution program is also activated^3^. Resolution of inflammation plays a crucial role in tendon healing^2^ as prolonged inflammation can impede healing^4^. Therefore, timely resolution is crucial for effective tendon healing and transition to subsequent repair phases, including proliferation and remodeling. Resolution of inflammation involves a complex interplay of various mediators and cellular processes, including mediators such as lipoxins and resolvins^5^. Additionally, anti-inflammatory cytokines like interleukin-10 (IL-10) contributes to resolution by inhibiting pro-inflammatory signaling pathways and facilitating transition of macrophages from M1 to M2 phenotypes^6^.

Anti-inflammatory drugs, like corticosteroids, exert their effects by resolving inflammation^7–9^. Corticosteroids can interfere with the inflammatory response^10^, but they can also influence the extracellular matrix and granulation of connective tissue^11^. Dexamethasone, a corticosteroid, has been previously investigated for its effects on tendon healing^12–14^. Earlier studies suggest that the effect of dexamethasone can vary based on factors such as timing, dosage, and the phase of tendon healing^13^. Recent research indicates that administering dexamethasone between days 7–11 post-injury, at a dosage of 0.1 mg/kg, can significantly improve the material properties of healing tendons while minimizing adverse effects, indicating a dose- and time-dependent response. Additionally, there is an interaction between dexamethasone and loading during tendon healing, with benefits observed even under partial unloading conditions (i.e. mild loading). Albeit the treatment effect appears to be more pronounced in fully loaded animals^12–14^. Dexamethasone has been found to modify the immune cell composition after an Achilles tendon injury, and result in a matrix with better organization, but further mechanistic insights remain unexplored. Particularly on the interaction between loading and dexamethasone.

The cells within the Achilles tendon can perceive mechanical forces and convert them into biochemical signals^15^. This process, known as mechanotransduction, enables cells to sense and adapt to varying loading conditions, resulting in changes in gene and protein expression^16^. Normally, mechanical forces play a crucial role in maintaining the physiological properties of tendons^15^. Unlike the gradual response of an intact tendon to forces, the healing tendon exhibits an immediate adaptive response^17^. Furthermore, the magnitude of loading influence the mechanical properties of the tendon differently: mild loading typically preserves tissue integrity, whereas unrestricted cage activity induces microdamage and alters the inflammatory response^18^. Unrestricted cage activity, which is considered physiological loading for an intact tendon, can cause small bleedings in newly formed tendon tissue during healing and is thus considered heavy loading in this context^18^. Consequently, the level of loading applied to the healing tendon triggers distinct mechanisms: mild loading primarily engages mechanotransduction, while heavy loading involves both mechanotransduction and microdamage.

Previous studies have investigated the main morphological and structural outcomes of dexamethasone on tendon healing models^12–14^. However, combined influence of loading magnitude and dexamethasone treatment may yield diverse molecular mechanism outcomes. We have previously observed distinct gene responses related to inflammation and the extracellular matrix under different loading conditions^18^﻿. Additionally, improved extracellular matrix orientation in healing tendons has been described after dexamethasone treatment^14^. Building on these findings, we hypothesized that dexamethasone treatment, modulated by the level of loading applied to the healing, will result in altered levels of markers associated with resolution and extracellular matrix composition. Therefore, this study aimed to investigate gene and protein changes following dexamethasone treatment under heavy or mild loading conditions in a rat model, as well as to examine the direct response of dexamethasone on human tendon cells in vitro*.*

## Results

### The effect of dexamethasone on resolution differs with load magnitude

**Figure 1:**
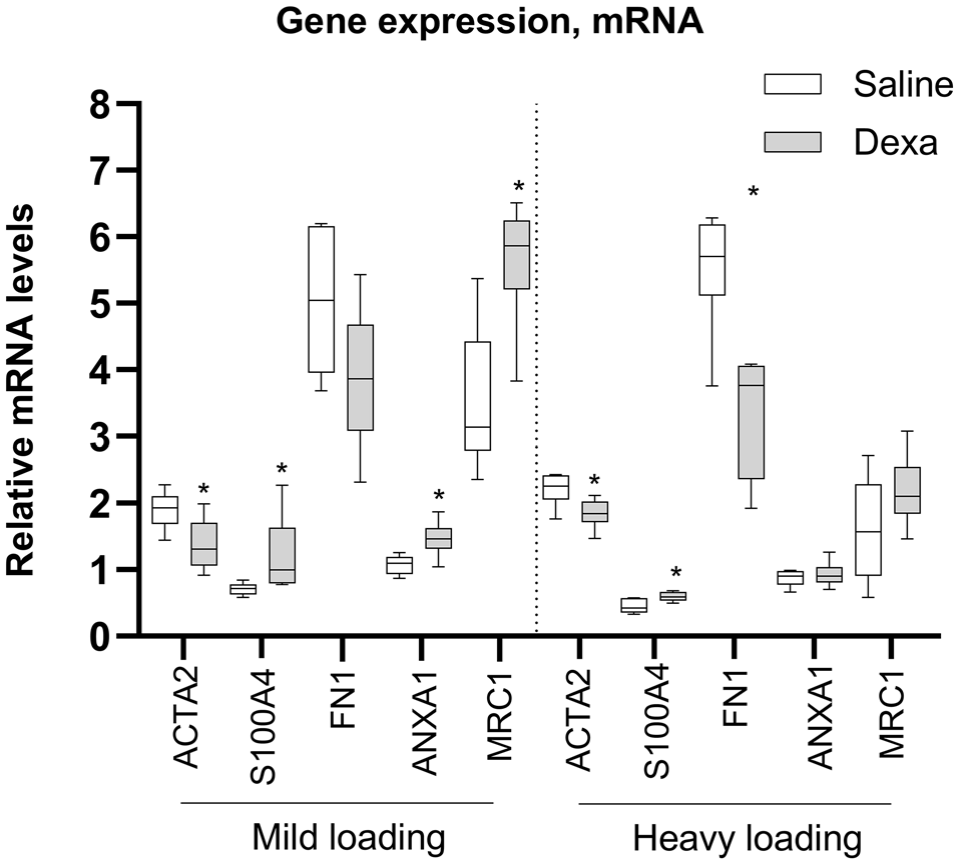
mRNA levels in healing tendons, 12 days post-injury. Relative mRNA levels are shown for saline (white boxes) and dexamethasone (Dexa, 0.1 mg/kg (grey boxes), under mild and heavy loading condition. *n* = 6 for each group. The mRNA levels are normalized to a geometric mean of three reference genes. *means *p* < 0.05.

**Figure 2:**
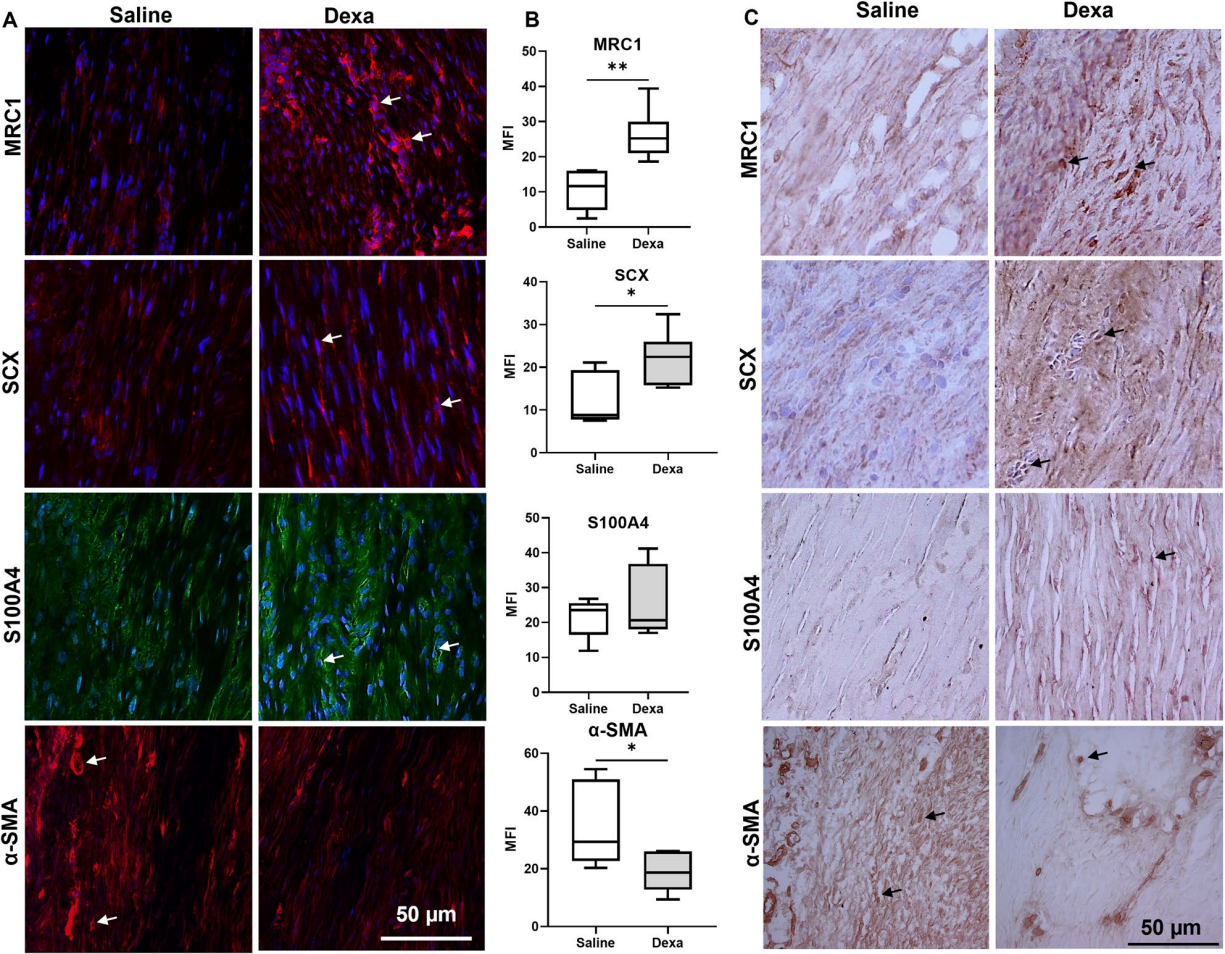
Immunofluorescence and Immunohistochemical staining in mildly loaded tendon tissue. (**A**) Immunofluorescence staining of MRC1 (red), SCX (red), S100A4 (green), and αSMA (red) in mid substance healing tendon tissue. DAPI (blue) was used as a nuclear staining of cells. (**B**) Quantification of the immunofluorescence staining, presented as mean and SEM in saline (*n* = 6) and dexa (*n* = 6) treated samples. All images were taken with same magnification, i.e. 200 × and at least 2 images were quantified following the same area. * indicates *p* ≤ 0.05; **p ≤ 0.01. (**C**) Representative histological staining of tendon tissues stained with DAB whereas MRC1, SCX, S100A4, and αSMA were detected in tendon inflammatory cells and tenocytes. Scale bars: 50 μm.

Healing tendons subjected to heavy loading showed no alterations in resolution related genes after dexamethasone treatment. Whereas mild loading condition, along with dexamethasone treatment, led to higher mRNA levels for several resolution related genes (Table 1, Fig. 1). Dexamethasone treatment increased the mRNA levels for annexin 1 (ANXA1, 37% higher, *p* = 0.01), mannose receptor C-type 1 (MRC1, 60% higher, *p* = 0.005), podoplanin (PDPN, 24% higher, *p* = 0.03), and prostaglandin E synthase (PTGES, 42% higher, *p* = 0.008). mRNA levels for TGF beta 1 (TGFB1), arachidonate 15-lipoxygenase (ALOX15), CD68, chemokine-like receptor 1 (CMKLR1), formyl peptide receptor 2 (FPR2), interleukin 6 (IL-6), and interleukin 10 (IL10) showed no statistical difference compared to saline. MCR1 expression was also increased in the dexamethasone group on a protein level (Fig. 2).

**Table 1:** Gene expression data, 12 days post-operatively, from heavy and mildly loaded animals, treated with saline or dexamethasone (Dexa, 0.1 mg/kg).

Gene	Heavy Loading					Mild loading			
	Saline Mean ± SD	Dexa Mean ± SD	*P* value	%		Saline Mean ± SD	Dexa Mean ± SD	*P* value	%
COL1A1	25 ± 9.0	25 ± 8.3	0.822	0		21 ± 4.3	21 ± 4.8	0.902	0
COL3A1	46 ± 9.1	51 ± 28	0.878	11		38 ± 5.1	50 ± 9.4	**0.028**	32
COL5A1	46 ± 23	59 ± 58	0.886	28		18 ± 3.4	21 ± 5.8	0.311	17
EDN1	2.7 ± 1.7	2.8 ± 0.89	0.711	4		2.0 ± 0.83	1.7 ± 0.24	0.659	− 15
ELN	7.2 ± 6.4	6.1 ± 1.6	0.730	− 15		4.4 ± 1.2	5.0 ± 1.9	0.597	14
TNC	14 ± 4.4	6.1 ± 2.1	**0.001**	− 56		7.5 ± 2.4	5.4 ± 1.0	0.160	− 28
FN1	5.5 ± 0.91	3.3 ± 0.91	**0.005**	− 40		5.0 ± 1.2	3.9 ± 1.0	0.104	− 22
LOX	2.4 ± 0.69	2.8 ± 0.64	0.302	17		2.6 ± 0.52	3.7 ± 1.1	0.069	42
SCX	110 ± 40	93 ± 81	0.411	− 11		36 ± 5.8	36 ± 12	0.795	0
S100A4	0.45 ± 0.10	0.59 ± 0.070	**0.018**	50		0.71 ± 0.90	1.2 ± 0.57	**0.023**	71
ACTA2	2.2 ± 0.25	1.8 ± 0.22	**0.026**	− 18		1.9 ± 0.28	1.4 ± 0.39	**0.024**	− 26
TGFB1	1.7 ± 0.46	1.7 ± 0.80	0.606	0		1.5 ± 0.23	1.7 ± 0.31	0.212	13
ALOX15	9.2 ± 8.2	23 ± 21	0.077	150		9.6 ± 4.5	16 ± 9.3	0.166	67
ANXA1	0.87 ± 0.13	0.93 ± 0.19	0.597	0		1.1 ± 0.14	1.5 ± 0.27	**0.010**	37
CD68	1.7 ± 0.57	1.8 ± 0.15	0.599	6		2.7 ± 0.72	3.3 ± 0.70	0.156	22
CMKLR1	5.8 ± 2.6	6.3 ± 2.2	0.989	9		4.6 ± 1.8	5.0 ± 0.70	0.363	9
FPR2	2.6 ± 1.3	3.8 ± 3.3	0.539	46		6.0 ± 4.3	4.5 ± 1.0	0.785	-25
IL6	0.6 ± 0.47	0.3 ± 0.40	0.158	− 50		2.1 ± 2.9	0.8 ± 0.54	0.547	-62
IL10	20 ± 8.8	23 ± 13	0.748	15		28 ± 15	37 ± 15	0.290	32
MRC1	1.6 ± 0.78	2.2 ± 0.53	0.137	38		3.5 ± 1.1	5.6 ± 0.94	**0.005**	60
PDPN	2.7 ± 0.21	2.8 ± 0.70	0.772	4		3.8 ± 0.71	4.7 ± 0.66	**0.033**	24
PTGES	0.8 ± 0.49	1.2 ± 0.42	0.071	50		1.2 ± 0.28	1.7 ± 0.30	**0.008**	42

*p*-values are from Student’s *t*-tests. *n* = 6 for all, except for heavy loading animals in IL10 (*n* = 5, saline group) and FPR2 (*n* = 5 for saline and dexamethasone group). The samples for these genes were excluded due to high cycle threshold (CT) value and too large variation in the CT value for the duplicates. Percentage was calculated in relation to the saline group. Bold indicates a significant difference compared to saline.

### Dexamethasone treatment also alters tendon cell related markers

**Figure 3:**
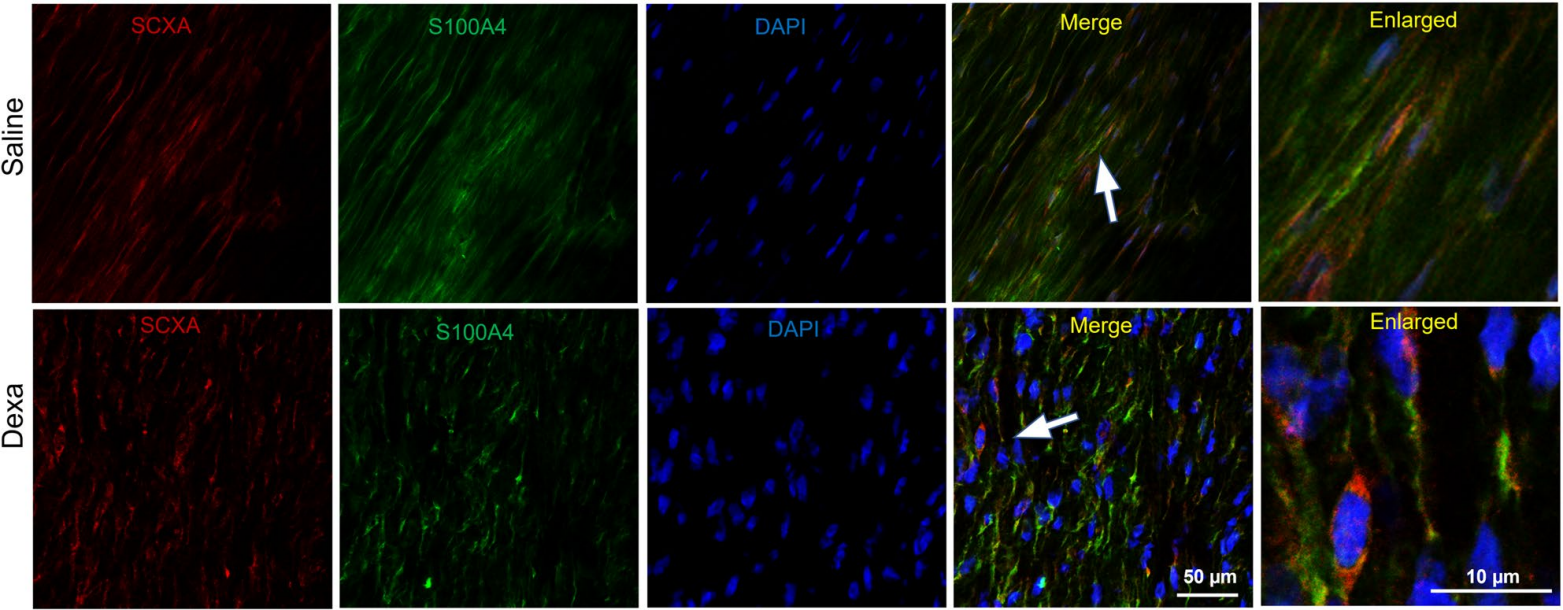
Visualization and co-localization of SCX with S100A4 in saline and dexamethasone (Dexa) treated rat tendons under a mild loading condition. Immunofluorescence staining of tenocytes showing partly co-localization of S100A4 (green) with SCX (red), a tenocyte marker, and DAPI (blue). Scale bars: 50 μm and 10 μm respectively. Images are collected from the midsubstance of the tendon.

Compared to saline, dexamethasone treatment doubled the mRNA levels for S100A4 (*p* < 0.05), while the levels for α-smooth muscle actin (ACTA2/αSMA) were reduced by 20% (*p* < 0.05, Fig. 1, Table 1). This was seen independent of the load magnitude. Protein levels of ACTA2/αSMA was also reduced after dexamethasone treatment while S100A4 showed no statistical difference between the groups (Fig. 2). An increase in the protein levels for scleraxis (SCX) after dexamethasone treatment in mildly loaded tendons was verified. There was also a co-expression in some of the cells between SCX and s100A4 (Fig. 3).

### Healing tendons with heavy loading show reduced levels of tenascin C and fibronectin after dexamethasone treatment

**Figure 4:**
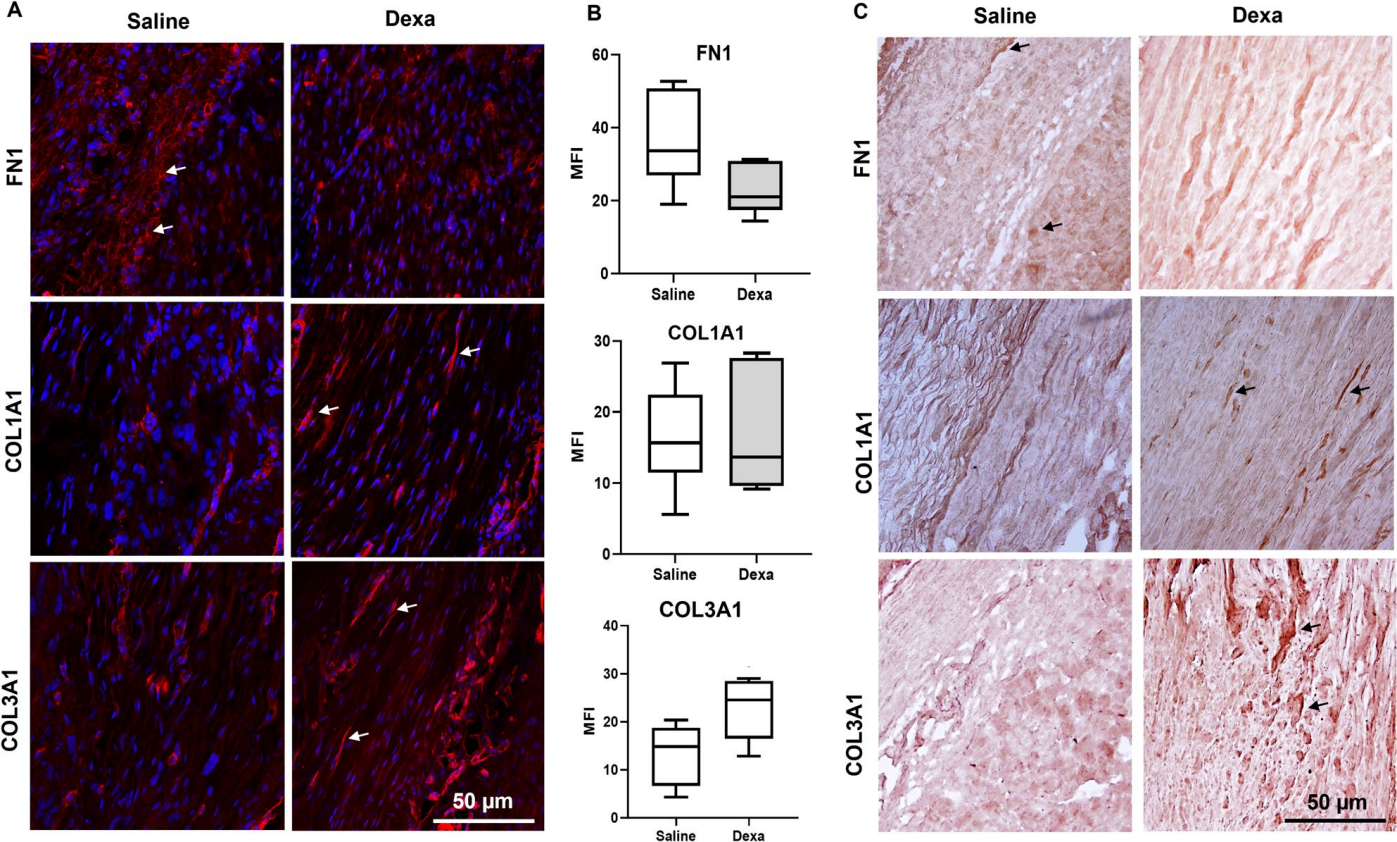
Immunofluorescence and Immunohistochemical staining in mildly loaded tendon tissue. (**A**) Immunofluorescence staining from the mid substance in the healing tendon tissue showing localization of FN1, COL1A1 and COL3A1 in red and DAPI staining in blue. **(B**) Quantification of the immunofluorescence staining presented as mean and SEM in saline (*n* = 6) and dexamethasone (*n* = 6) treated samples (all images were taken with same magnification i.e. 200 × and at least 2 images were quantified following the same area). (**C**) Representative histological staining of tendon tissue stained with DAB. Scale bars: 50 μm.

The mRNA levels for extracellular matrix related genes, fibronectin (FN1) and tenascin C (TNC) were reduced after dexamethasone treatment by 40% for TNC (*p* = 0.005) and 56% for FN1 (*p* = 0.001, Fig. 1, Table 1). The reduced mRNA levels were only seen in tendons subjected to heavy loads, and not in the tendons subjected to mild loads (no statistical difference). In contrast, mRNA levels for collagen type 3 (COL3A1) were 32% higher after dexamethasone treatment (*p* = 0.03) in mildly loaded tendons. The proteins levels for COL3A1 and FN tended to differ in the same direction but not significantly (*p* = 0.095, and *p* = 0.13 respectively, Fig. 4).

### Dexamethasone alters the cellular properties of tendon cells in vitro

**Figure 5:**
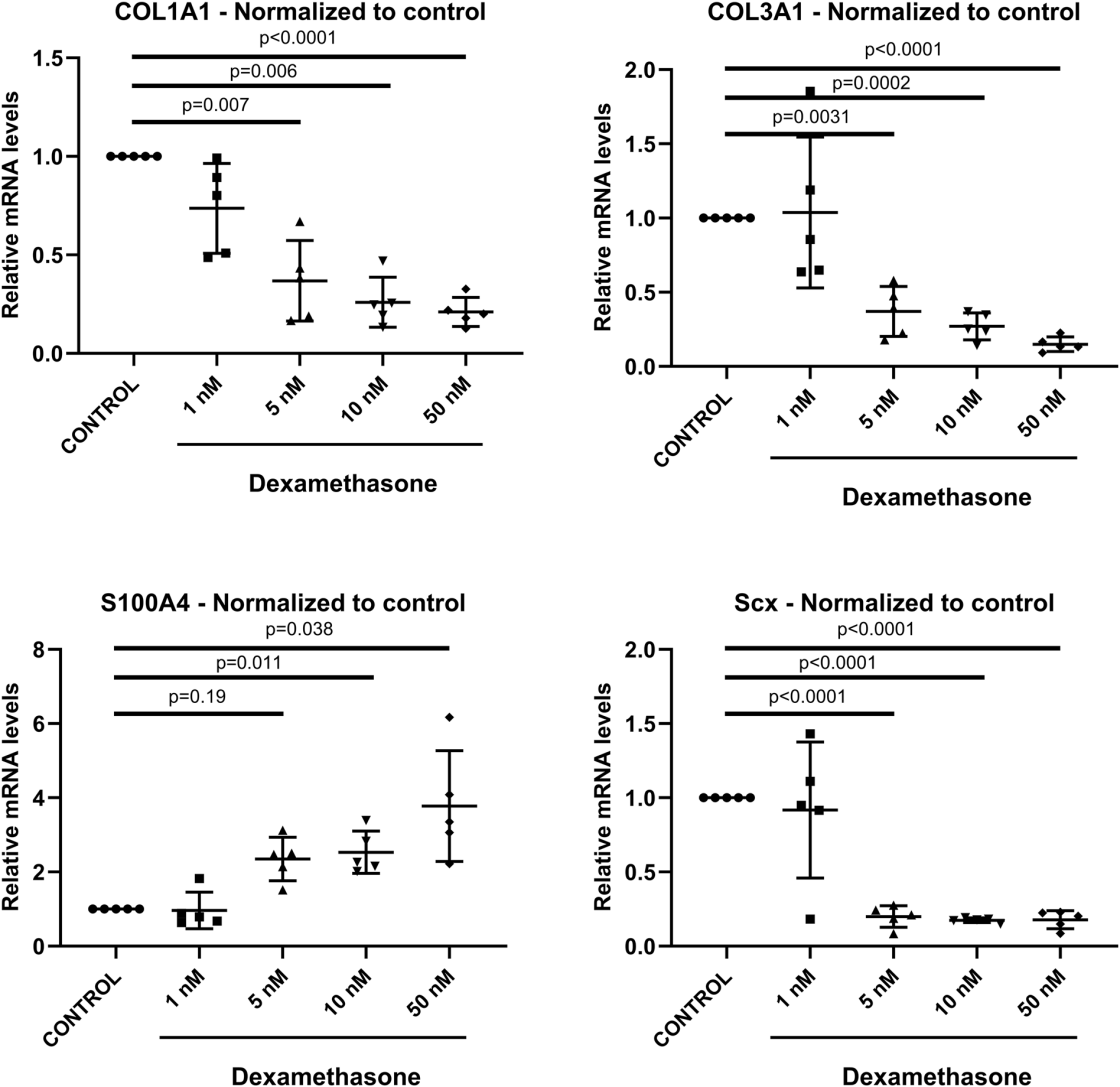
mRNA levels in tendon constructs with or without dexamethasone treatment 14 days post-seeding. The constructs were treated with dexamethasone (1, 5, 10, or 50 nM) or control. The mRNA levels for each gene were normalized to the reference gene YWHAZ and thereafter normalized to its control. *N* = 5 for each group.

The in vivo study indicated, not only an immunomodulatory effect of dexamethasone, but also suggested a possible effect on tendon cells. Therefore, an in vitro study was performed to verify an effect on tendon cells without any immunomodulatory response. Dexamethasone treatment increased the mRNA levels for S100A4 by 2.4–3.8-fold for the 10 and 50 nM group compared to control (*p* = 0.0015, Fig. 5, Table 2). In contrast, the mRNA levels for SCX were reduced to 0.2–0.17-fold by the same treatment (*p* = 0.0084). The levels for ACTA2/αSMA showed no statistical difference between dexamethasone treated constructs and controls (*p* = 0.38). Dexamethasone had an overall effect on Ki67 (*p* = 0.030), and the 1 nM group differed from controls, but there was no apparent dose–response effect.

**Table 2:** Gene expression data from tendon constructs 14 days post seeding, with or without dexamethasone treatment (1, 5, 10, and 50 nM).

Gene	Control	Dexamethasone concentration				*P*-value
		1 nM	5 nM	10 nM	50 nM	
COL1A1	1	0.74 ± 0.23	0.37 ± 0.20*	0.26 ± 0.13*	0.21 ± 0.073*	0.0001
COL3A1	1	1.0 ± 0.51	0.37 ± 0.17*	0.27 ± 0.091*	0.15 ± 0.050*	0.0081
LOX	1	2.0 ± 0.77	1.4 ± 0.58	1.3 ± 0.44	1.8 ± 0.63	0.095
TGFB1	1	1.5 ± 0.57	0.86 ± 0.28	0.95 ± 0.23	1.0 ± 0.30	0.055
SCX	1	0.92 ± 0.46	0.20 ± 0.073*	0.17 ± 0.016*	0.18 ± 0.060*	0.0084
S100A4	1	0.96 ± 0.49	2.4 ± 0.59*	2.5 ± 0.57*	3.8 ± 1.5*	0.015
TNMD	1	0.091 ± 0.032	0.072 ± 0.045	0.038 ± 0.0037	0.062 ± 0.0088	N/A
ACTA2	1	1.1 ± 0.26	1.2 ± 0.20	1.2 ± 0.13	1.2 ± 0.32	0.38
Ki67	1	1.2 ± 0.19*	0.68 ± 0.18	0.80 ± 0.17	0.94 ± 0.31	0.030

* Means significantly different from control.

The data is normalized to each control. *p*-values (*p*) are from repeated measured ANOVA. *n* = 5 for all, except for TNMD which have missing data due to low expression in the dexamethasone treated groups. Mean ± SD (standard deviation).

In contrast to the animal experiment, the in vitro study showed reduced mRNA levels after dexamethasone treatment for COL1A1 and COL3A1 compared to control (0.37–0.21-fold reduction for COL1A1 and 0.37–0.15-fold for COL3A1, *p* = 0.0001 and *p* = 0.008 respectively). Lysyl oxidase (LOX) and transforming growth factor beta 1 (TGFB1) showed no statistical difference between treated constructs and control.

### High doses of dexamethasone prevent tendon construct formation

**Figure 6:**
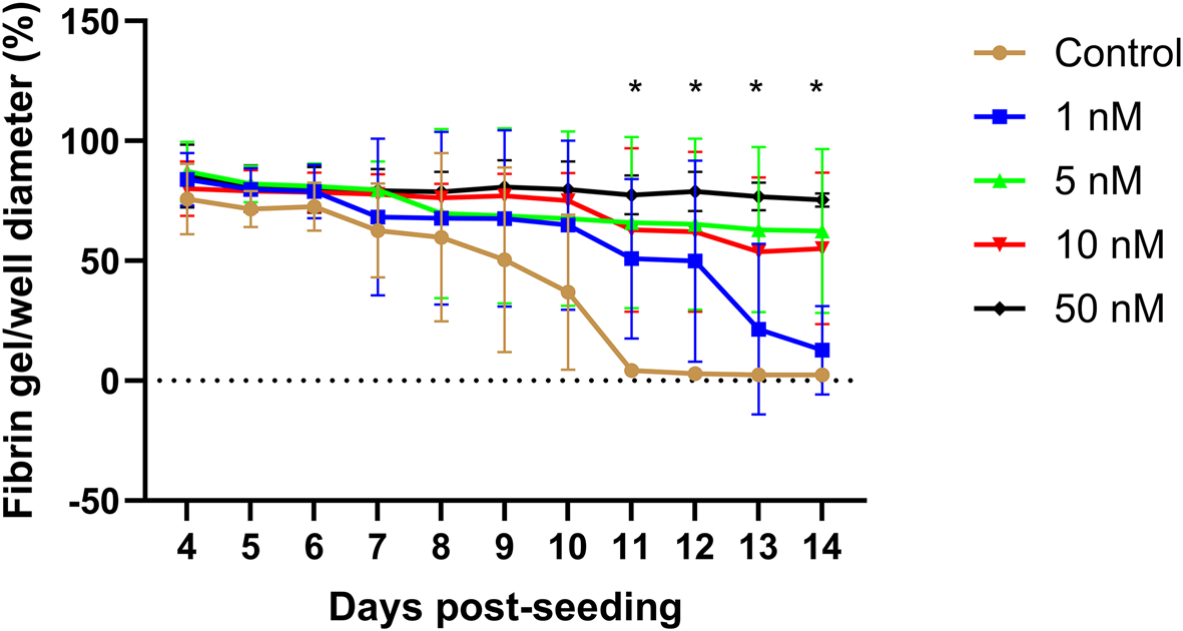
Fibrin gel contraction between day 4–14 post-seeding, with or without dexamethasone treatment (1, 5, 10, and 50 nM). The data are presented as percentage contraction in relation to the well, where 100% are complete covering of the well and 2–3% are fully contracted constructs. There were significant differences (*) between controls and dexamethasone treated constructs after day 11 (Except for 1 nM). The 5 nM group differed significantly from controls days 11, 12, 13, and 14 (*p* < 0.05). The 10 nM differed significantly from controls day 11 and 12 (*p* < 0.05), but not day 13 and 14 (*p* = 0.055/0.054). The 50 nM differed significantly from controls days 11, 12, 13, and 14 (*p* < 0.0001).

Image analysis of fibrin gel contraction showed a significant effect of time (*p* < 0.001 and treatment *p* < 0.03). There was also an interaction between time and treatment (*p* < 0.0001). The control group differed significantly from all dexamethasone treated groups, except for 1 nM, as at day 11 and 12 and from the 50 nM and 5 nM group at day 13 and 14 (Fig. 6). All gels in the control group were contracted and had formed tendon constructs at day 14 in contrast to the 50 nM group where none of the samples had formed construct. Four out of five gels did not contract in the groups treated with 5 nM or 10 nM.

## Discussion

This study shows that dexamethasone can trigger different gene responses depending on the in vivo loading of the healing tendon. The treatment effect in heavily loaded tendons appears to be related to altered extracellular matrix and cell-related markers, while the effect in mildly loaded tendons is also related to altered resolution of inflammation. Histologic analysis confirms altered levels of proteins related to extracellular matrix, cells and resolution. In vitro experiments also confirm a specific treatment effect on tendon cells with inhibited fibrin gel contraction, reduced mRNA levels of several tendon-related genes and increased mRNA levels of S100A4.

This study employed two distinct in vivo loading regimens, following previous research which revealed significant dexamethasone-induced alterations in material properties within heavily loaded tendons, with a smaller yet notable effect in mildly loaded tendons^13^. Additionally, investigations have demonstrated that intense loading shortly after tendon injury can induce a prolonged inflammatory response due to micro-damages within the tissue, while milder loading appears to influence mechanotransduction processes^18,19^. We have not measured the exact amount of loading in each group, but we considered cage activity to be heavy loading due to the occurrence of small bleedings in the early healing tissue^18^. Botox, on the other hand, will produce some loading, as observed in previous studies^20^. Yet, while cage activity is indeed high-loading relative to botox, it is not likely reflective of physiological high-loading. Our findings revealed varied treatment effects at the mRNA level depending on the degree of loading. Dexamethasone administration in heavily loaded tendons exhibited diverse effects on certain matrix-related genes, whereas in mildly loaded tendons, its impact was more associated with inflammation resolution. The results suggest a link between loading intensity, microdamage, inflammation, and tendon healing, thereby supporting the hypothesis that different loading conditions after dexamethasone treatment may activate other mechanisms and subsequently distinct gene responses after tendon healing.

In a previous study, no reduction in leukocyte proportion was observed in tendon tissue during healing following dexamethasone treatment, indicating alternative treatment effects^14^. After injury, the resolution program begins when tissue inflammation starts^3^. We hypothesized that dexamethasone is involved in this process as a “base” for the final resolution of inflammation. Although this process was not complete 12 days post-injury, it continued further in the saline-treated group. This assumption led us to postulate that genes associated with resolution would be upregulated in the dexamethasone-treated group. While not statistically significant, mRNA levels of the pro-resolving protein ALOX15 were observed to be 150% higher in the fully loaded dexamethasone-treated group compared to controls. Additionally, an increase in MRC1, a macrophage mediator marker, was noted in dexamethasone-treated rats. ANXA1, known as a pro-resolving marker, followed the similar pattern to MRC1. Both ANXA1 and macrophages play integral roles in apoptotic cell clearance within the tissue^21^, thus influencing resolution. ANXA1´s mechanism in enhancing resolution involves accelerating apoptosis^22^, and it has also been implicated in tissue repair processes^23^. Our findings suggest that termination of inflammation through dexamethasone treatment could explain the positive mechanical findings achieved in previous studies^13^. However, it is important to note that we did not conduct any long-term experiments to investigate the sustained effects of dexamethasone treatment, which represents a limitation of our study.

In both intact and healing tendons, various cell types are present. A commonly used marker for tendon fibroblasts is SCX^24^, but recent research has shown that both myofibroblasts (αSMA+ cells) and S100A4+ fibroblasts are two additional cell populations within the mature tendon^24,25^. Importantly, all these cell populations play roles in tendon healing processes. Our study demonstrates that dexamethasone treatment leads to decreased mRNA and protein levels of ACTA2/αSMA, while increasing mRNA levels for S100A4. The reduction in αSMA levels aligns with findings from studies on corticosteroids, and wound healing, as well as studies on airways in mice^26,27^. The similarity between the behavior of skin fibroblasts in wound healing and tendon fibroblasts in tendon healing is expected due to the resemblance of repair processes in these tissues. Tendon injuries typically heal in a fibrotic manner, characterized by excessive and disorganized deposition of extracellular matrix^28^. Both Myofibroblasts (αSMA+ cells) and S100A4+ cells are highly active in fibrosis^29^. While S100A4 serves as a marker for tenocytes in intact tendons and granulation tissue, it is also critical driver of fibrosis and fibrotic tendon healing^30^. Although our investigation did not explore the specific roles of S100A4 (such as changes in tendon cell phenotype or its role in fibrotic healing), we did observe an effect of dexamethasone on S100A4 levels both in vivo and in vitro.

Dexamethasone treatment resulted in reductions of the extracellular matrix-related components, fibronectin, and tenascin C. Fibronectin has previously been shown to increase rapidly after injury and plays a role in fibrosis^31,32^. Similarly, tenascin C has been shown to facilitate the early stages of myocardial repair by promoting the recruitment, migration, and differentiation of myofibroblasts^33^. The decrease observed in αSMA and FN1 suggest a potential reduction in fibrosis or fibrotic healing due to dexamethasone treatment. Previous studies utilizing the same animal model have demonstrated that dexamethasone treatment improves the material properties of tendons^12,13^.

In this study, we employed systemic administration of dexamethasone, which differs from its use in patients. However, even local administration can lead to systemic effects, including granulocytosis and lymphopenia^13^. High doses of dexamethasone have been shown to influence stress levels and result in adverse effects, such as weight loss and altered homeostasis. The dosage utilized in this study has previously demonstrated a beneficial effect on tendon healing with minimal adverse effects. Notable, we did not observe any significant differences in weight between the treatment groups.

In our in vitro experiments, we observed changes in mRNA levels for several genes. SCX exhibited a dose-dependent decrease, consistent with previous studies on tendon cell cultures and dexamethasone treatment^34^ Dexamethasone has been reported to inhibit the differentiation of tendon stem cells into tenocytes by suppressing the SCX gene^35^, as evident by higher expression levels in the controls compared to all dexamethasone-treated groups, suggesting a potential alteration in cell phenotype. TNMD expression is typically lower when cultured outside a tendon construct^36^ and glucocorticoids further reduce these levels^34^. Contrary to TNMD and SCX, S100A4 expression was elevated following dexamethasone treatment. Increased S100A4+ cells have previously been identified as a subpopulation of tendon cells involved in tendon healing^24,25^. Increased S100A4 expression has also been associated with TGFB1-induced fibroblast activation in skin fibroblasts^37^, resulting in upregulated production of αSMA and type I collagen^38^. However, in our study, neither TGF1 nor αSMA expression was affected by dexamethasone treatment, while collagen type I and III were both downregulated. The effects of dexamethasone on S100A4 are not extensively documented, but consistent with our findings, a previous study demonstrated increased S100A4 expression in osteoblasts after corticosteroid treatment^39^. Corticosteroids have been proposed to exert direct effects on mesenchymal cells, reducing their proliferation^35,40^. While proliferation was not directly assessed in our study, regular monitoring of the cells revealed no apparent differences under light microscopy.

Our hypothesis was that cell-mediated contraction is driven by myofibroblasts, as these cells have been implicated in both cell-mediated tendon contraction^41^ and in vitro models of tendon healing^42^. Additionally, corticosteroids have been shown to inhibit the accumulation of myofibroblasts in the airways of mice^43^. Our experiments revealed that dexamethasone inhibited fibrin gel contraction, yet had no discernible effect on mRNA levels for ACTA2/α-SMA. However, it is worth noting that we only assessed gene expression at a single time point for all cells, irrespective of the day each construct was formed. This limitation provides only a snapshot of gene expression, which fluctuates over time as seen in previous research where myofibroblast differentiation was observed as early as 1–2 days before the onset of wound contraction^42^.

## Conclusion

Dexamethasone appears to facilitate tendon healing not only by resolving inflammation but also by mitigating fibrotic healing. Our findings suggest that dexamethasone may improve tendon healing beyond its immunomodulatory effects. Nonetheles, further investigations are needed to better understand the process behind dexamethasone and tendon healing, particularly in contexts involving mild loading. If equivalent outcomes can be replicated in clinical settings using systemic dexamethasone, which is both fast-acting and cost-effective, it could pave the way for innovative approaches to treating Achilles tendon injuries.

## Materials and methods

### Study design

This study was performed using a rat model for Achilles tendon healing and a 3D cell culturing model using primary human tendon cells. All evaluations followed a blinded approach (Table 3).

**Table 3:** Experimental design. In vivo experiment used a rat model for Achilles tendon healing with heavy or mild in vivo loading.

	Group	Interventions	Analyzes
In vivo – rat model	Heavy loading + Saline (Control)	Tendon transection (D0) Saline injections (D7-11) Euthanization (D12)	qPCR (*N* = 6)
	Heavy loading + Dexamethasone 0.1 mg/kg	Tendon transection (D0) Dexa injections (D7-11) Euthanization (D12)	qPCR (*N* = 6)
	Mild Loading + Saline (Control)	Botox injections (D-4) Tendon transection (D0) Saline injections (D7-11) Euthanization (D12)	qPCR (*N* = 6) IHC/IF (*N* = 6)
	Mild Loading + Dexamethasone 0.1 mg/kg	Botox injections (D-4) Tendon transection (D0) Dexa injections (D7-11) Euthanization (D12)	qPCR (*N* = 6) IHC/IF (*N* = 6)
In vitro – human tendon cells	Control	Cell seeding (D0) Imaging (D4-14) Sample collection (D14)	qPCR (*N* = 5) Imaging (*N* = 5)
	Dexamethasone 1, 5, 10 or 50 nM	Cell seeding (D0) Dexa treatment (D2-14) Imaging (D4-14) Sample collection (D14)	qPCR (*N* = 5) Imaging (*N* = 5)

The Achilles tendon was transected at day 0 and animals were treated with saline or dexamethasone (0.1 mg/kg) from day 7–11. All animals were euthanized at day 12 and tendons were used for qPCR, immunohistochemistry, and immunofluorescence (IHC/IF). In vitro experiment used a 3D cell culturing model with primary human tendon cells treated with or without different doses of dexamethasone (1–50 nM). D means day of intervention.

### Animals and housing

A total of 36 female Sprague–Dawley rats, Specific Pathogen Free (Taconic Biosciences), weighing on average 207 g (SD17) were randomly divided by lottery into four groups: (1) Heavy loading + saline, (2) Heavy loading + dexamethasone, (3) Mild loading + saline, (4) Mild loading + dexamethasone (Table 1). The group sizes are based on experience using this model. The Regional Ethics Committee for animal experiments in Linköping, Sweden, approved all procedures (Ref. ID1424). Rats were placed two and two and acclimated for two weeks before the experiment started. The room had a standard humidity (55%) and temperature (22 °C), and a 12-h light–dark cycle (light from 7 am to 7 pm). Water and food pellets were offered ad libitum.

### Botox injections to reduce loading

Rats with mild loading received Botulinum toxin injections (Botox, Allergan, Irvine, USA) in the calf muscles four days prior to surgery. Injections were performed under anesthesia with isoflurane gas (Forene, Abbot Scandinavia, Solna, Sweden). A total of 3U of Botox (0.06 mL/animal) was injected into the right hind leg, targeting the gastrocnemius lateralis, gastrocnemius medialis, and soleus muscles (1U Botox/muscle). Botox effectiveness was confirmed in all animals before tendon transection by visual inspection of the loading pattern on the paw.

### Achilles tendon transection

The surgery day was counted as day 0, and rats were anesthetized with isoflurane gas. The right hind leg was shaved and cleaned with chlorhexidine ethanol. The tendon complex was exposed through a minor transverse skin incision lateral to the Achilles tendon. The plantaris tendon was completely removed to avoid interference during healing and sample collection. The Achilles tendon was transected in the mid-tendon portion and left to heal unsutured, while the skin was closed. Antibiotic was given preoperatively (25 mg/kg, oxytetracycline, Engemycin, Intervet, Netherlands) to avoid post-operative infection. Analgesic was given subcutaneously pre- and post-operatively every 8–12 h for 48 h (0.045 mg/kg, buprenorphine, Temgesic, Indivior Europe Limited, Ireland). Rats received systemic subcutaneous injections of saline solution 0.9% as control or dexamethasone (0.1 mg/kg, Dexaject; Dopharma Research B.V.) from day 7–11 at 3.30 pm. The healing tendons were collected 12 days post-operatively. The rats were anesthetized with isoflurane, and the mid-part of the newly formed tendon was collected and snap frozen in liquid nitrogen or were collected for histology. The rats were euthanized by a cardiac pentobarbital injection and the samples were stored at -80º until analysis. No animals had to be excluded from the study.

### Cell extraction

Human tendon fibroblasts were isolated by collagenase digestion from semitendinosus tendons from patients (*n* = 5) undergoing anterior cruciate ligament reconstructive surgery as previously described^36^. The experiments were approved by the the Regional Ethical Review Board in Linköping, Sweden (2015/408-31) and patients gave written informed consent. Cells were seeded in flasks and cultured to confluence in DMEM/F12 supplemented with 10% fetal bovine serum (FBS) and 1% Penicillin–Streptomycin.

### Construct formation

Tendon constructs were assembled as previously described^36^. Six-well plates were coated with silicon (SYLGARD, Dow-Chemicals) and two silk sutures (0.5 cm, Ethicon) were pinned downed as anchor points (15 mm apart). 250 000 cells (passage 2–5) were mixed into a fibrin gel and quickly spread in each well. The fibrin was left to set before it was covered with cell culture supernatant (DMEM/F12 supplemented with 10% FBS, 0.2 mM L-ascorbic acid 2-phosphate, 0.05 mM L-Proline and 1% Penicillin–Streptomycin). Cell culture supernatant was replaced every second to third day and adhesions to the side of the well were detached 2 days post-seeding using a fine pipette tip to allow gel contraction. Cells were treated with dexamethasone from day 2–14 post-seeding. Four different concentrations were used; 1, 5, 10, or 50 nM. The drug was diluted in cell culture supernatant and controls were treated with 1 mM of ethanol. DEXAJECT® includes ethanol and 1 mM corresponds to the levels in the 50 nM group. All samples were harvested 14 days post-seeding.

### Image acquisition and analysis

Fibrin gel contraction was measured on images taken day 4–14 post-seeding by a mobile phone on a standardized distance (11 cm) above the 6-well plate. The vertical diameter of the fibrin gel was measured at its widest point and normalized to the vertical diameter of the well in ImageJ (1.51 k National Institutes of Health, USA). Results are presented as a percentage of contraction of the well.

### RNA extraction

#### Tissue

Samples (< 100 mg) were pulverized one by one by a tungsten ball using in a nitrogen cooled container using a Mixer Mill (Retsch, Germany) at 2,600 rpm for 45 s. The process was repeated if samples were not fully homogenized. 1000 µl of TRIzol (Life Technologies, Gibco BRL) was added to each sample and thawed at room temperature. RNA was separated by adding chloroform, followed by centrifugation (12000xg for 15 min at 4 ºC). The aqueous phase was collected in new tubes, and 70% ethanol was added. A RNeasy Total RNA mini kit (Qiagen, Germany) was used for further RNA extraction according to instructions.

#### Tendon constructs

Samples were rinsed with PBS and collected in tubes containing 1 ml TRIzol, 5 stainless steel beads (2.3 mm in diameter), and 5 silicon-carbide sharp particles (1 mm in diameter) for mechanical disruption (BioSpec Products Inc, USA). Samples were snap frozen and stored in -80 °C until analysis. After thawing samples on ice, they were homogenized at 30 Hz, for 15 s using TissueLyser (Qiagen). Disruption was repeated 2–4 times with 2 min on ice in between the repetitions. RNA was separated by adding 100 µl bromo-chloropropane (Molecular Research Centre, USA) followed by centrifugation (12000xg for 15 min at 4 ºC). The aqueous phase was collected and mixed with isopropanol (equal parts) and 4 µl of glycogen (20 µg/µl, Invitrogen, USA). The pellet was washed repeatedly with ethanol and dissolved in RNAse-free water. RNA concentration, purity, and quality were verified with Nanodrop ND-1000 (NanoDrop Technologies, USA) and RNA 6000 Nano kit (Agilent Bioanalyzer Technologies, Germany). All samples had an RIN value above 5 and were used for further analysis.

### cDNA synthesis and quantitative real-time PCR

Total RNA (1.5 μg) was converted to cDNA using the High Capacity cDNA Reverse Transcription Kit (Life Technologies, UK). Primers for genes related to extracellular matrix, resolution, and tendon cell markers were bought from Applied Biosystems (Supplementary table 1). CYPA, RPLPO, and UBC were tested as internal controls for the rat tissue samples and each sample was normalized to a geometric mean of these three genes. GUSB, RPLP0, and YWHAZ were tested as internal controls for the tendon constructs samples and YWHAZ was chosen as it was stably expressed between the groups.

Fast PCR Master Mix (Life Technologies) was used for amplification (15μL/reaction) and the samples were analyzed in duplicates. 3 samples were not included in the final analysis in the high loaded group for IL10 or FPR2. The samples for these genes were excluded due to high cycle threshold (CT) value and too large variation in the CT value for the duplicates. Quantification was calculated using a standard curve from universal rat reference gene (C.N. #QS0641, Life Technologies) or universal human reference gene (C.N. #QS0639). Reactions with no reverse transcription and no template were added as negative controls. Samples were analyzed with the 7.500 software, version 2.3 (Life Technologies).

### Immunohistochemistry

Tendons were embedded in OCT, snap-frozen, and sectioned longitudinally (7 μm) using a Cryostat Microtome (Leica, CM1950, Heidelberg, Germany). Samples were quickly stored at -80 ºC for further immunostaining. Anti-Rabbit HRP-DAB Cell & Tissue Staining Kit (catalogue #CTS005, R&D Systems, USA) was used for blocking, secondary antibody and chromogenic staining according to instructions^44,45^.

The primary antibodies used were: rabbit monoclonal [EPR5368] to alpha smooth muscle Actin (αSMA, 1:500, C.N #ab124964, Abcam, UK), and rabbit polyclonal to Fibronectin (FN1, 1:100, C.N #ab2413), Collagen type I (1:100, C.N#34,710), Collagen type III (1:100,C.N#7778), scleraxis (1:100, C.N # ab58655), and Mannose Receptor (1:250, C.N # ab64693), and goat polyclonal to S100A4 (1:100; C.N #ab58597). The primary antibodies were incubated overnight at 4 ºC. Washing steps and incubations with primary antibody were performed in PBS with 0.1% saponin. Tissue sections were blocked with 5% normal goat serum after the primary antibody and followed by a biotinylated anti- mouse secondary antibody (R&D Systems, USA). Tissue sections were developed by 3,3′-Diaminobenzidine (DAB) and counterstained with Mayer’s Hematoxylin (Sigma Aldrich, USA) before image visualization, using a standard light microscope.

### Immunofluorescence

Staining was performed as described^44,45^ with some modification. Briefly, after the primary antibody incubation (as described above) and washing in PBS (2 × 5 min). A mixture of biotinylated secondary antibodies including anti-rabbit conjugated with Alexa Fluor 594, and anti-goat conjugated with Alexa Fluor 488 (1:500 dilution in PBS-0.1% saponin) was incubated for 60 min. DAPI (4,6-diamidino-2-phenylindole, Invitrogen, USA) staining was performed for nuclei visualization.

### Image acquisition and analysis

Digital images were captured by a confocal and/or a super resolution microscope (Upright Zeiss LSM700 or LSM710, Carl Zeiss, Germany) and analyzed with ZEN 2009 software (LSM 710; Carl Zeiss). All pictures were taken at original magnifications of 200x, 400 × or 630 × with oil objective and analyzed with ImageJ software (Fiji ImageJ 1.52i, USA).

### Statistics

Results were analyzed using SPSS software version 21, and graphs were created using GraphPad Prism version 9. Gene expression data were log-transformed to obtain similar variances between the groups. Independent t-tests were used in the animal experiment to compare dexamethasone treated and saline group (controls) within each loading condition. Protein quantification data was analyzed with Mann–Whitney U tests. The in vitro data was first normalized to each control and thereafter analyzed by repeated measured ANOVA with Dunnets multiple comparisons test. The gel concentration data was analyzed with a 2-way ANOVA with time and treatment as independent variables. Results were considered significant when *p* < 0.05. Potential confounders were minimized by random locations of the different groups in the animal facility, a random order of surgery and euthanization from the different groups. The surgeon was kept blinded from group allocation during surgery and euthanization and analyzes were performed blinded from group allocation until statistical analysis.

### Supplementary Information

Supplementary Table 1.

## Data Availability

The datasets used and/or analyzed during the current study available from the corresponding author on reasonable request.
