# Supplementary material for: Dexamethasone treatment influences tendon healing through altered resolution and a direct effect on tendon cells
Source: Sci Rep. 2024 Jul 3;14:15304. doi: 10.1038/s41598-024-66038-5 (PMC11222440; doi:10.1038/s41598-024-66038-5)
Supplement: Supplementary file 1 — Supplementary Table 1. [file 41598_2024_66038_MOESM1_ESM.docx]

**Supplementary table 1**. Primers used for qRT-PCR. All primers were purchased from Applied Biosystems.

|  |  | **Gene symbol** | **Gene name** | **Ref. Sequence** |
| --- | --- | --- | --- | --- |
| **Rat assays** | ACTA 2 | Actin, Alpha 2, Smooth Muscle | NM_031004.2 | Rn01759928_g1 |
|  | COL1A1 | Collagen Type I, Alpha 1 | NM_053304.1 | Rn01463848_m1 |
|  | COL3A1 | Collagen Type III Alpha 1 | NM_032085.1 | Rn01437681_m1 |
|  | COL5A1 | Collagen Type V Alpha 1 | NM_134452.1 | Rn00593170_m1 |
|  | EDN1 | Endothelin 1 | NM_012548.2 | Rn00561129_m1 |
|  | ELN | Elastin | NM_012722.1 | Rn01499782_m1 |
|  | FN1 | Fibronectin 1 | NM_019143.2 | Rn00569575_m1 |
|  | LOX | Lysyl Oxidase | NM_017061.2 | Rn01491829_m1 |
|  | SCX | Scleraxis BHLH Transcription Factor | NM_001130508.1 | Rn01504576_m1 |
|  | S100A4 | S100 Calcium Binding Protein A4 | NM_012618.2 | Rn01451938_m1 |
|  | TGFB1 | Transforming Growth Factor, Beta 1 | NM_021578.2 | Rn00572010_m1 |
|  | TNC | Tenascin C | NM_053861.1 | Rn01454948_m1 |
|  | ALOX15 | Arachidonate 15-Lipoxygenase | NM_031010.2 | Rn00696151_m1 |
|  | ANXA1 | Annexin A1 | NM_012904.2 | Rn00563742_m1 |
|  | CD68 | Cd68 molecule | NM_001031638.1 | Rn01495634_g1 |
|  | CMKLR1 | Chemokine-Like Receptor 1 | NM_022218.2 | Rn00573616_s1 |
|  | FPR2 | Formyl Peptide Receptor 2 | XM_001073508.4 | Rn03037051_gH |
|  | IL6 | Interleukin 6 | NM_012589.2 | Rn01410330_m1 |
|  | IL10 | Interleukin 10 | NM_012854.2 | Rn01483988_g1 |
|  | MRC1 | Mannose receptor, C type 1 | NM_001106123.2 | Rn01487342_m1 |
|  | PDPN | Podoplanin | NM_019358.1 | Rn00571195_m1 |
|  | PTGES | Prostaglandin E Synthase | NM_021583.3 | Rn00572047_m1 |
|  | CYPA | Peptidylprolyl Isomerase A | NM_017101.1 | Rn00690933_m1 |
|  | RPLP0 | Ribosomal Protein, P0 | NM_022402.2 | Rn00821065_g1 |
|  | UBC | Ubiquitin C | NM_017314.1 | Rn01789812_g1 |
| **Human** **assays** | COL1A1 | Collagen type I alpha 1 | NM_000088 | Hs00164004_m1 |
|  | COL3A1 | Collagen type III alpha 1 | NM_000090 | Hs00943809_m1 |
|  | LOX | Lysyl Oxidase |  | Hs00942483_m1 |
|  | SCX | Scleraxis bHLH transcription factor | NM_001080514 | Hs03054634_g1 |
|  | S100A4 | S100 calcium binding protein A4 | NM_002961 | Hs00243202_m1 |
|  | TNMD | Tenomodulin | NM_022144.2 | Hs00223332_m1 |
|  | ACTA2 | Actin alpha 2, smooth muscle | NM_001141945.2 | Hs00426835_g1 |
|  | TGFB1 | Transforming growth factor beta 1 | NM_000660.5 | Hs00998133_m1 |
|  | MKI67 | Marker of proliferation Ki-67 | NM_001145966.1 | Hs04260396_g1 |
|  | YWHAZ | Tyrosine 3-monooxygenase/tryptophan 5-monooxygenase activation protein zeta | NM_001135699 | Hs01122445_g1 |
